# Data entry checking - is it necessary?

**DOI:** 10.1186/1745-6215-14-S1-P62

**Published:** 2013-11-29

**Authors:** Gladys McPherson

**Affiliations:** 1 University of Aberdeen, Aberdeen, Scotland, UK

## 

Whether or not to incorporate data entry checking into your Data Management Plan and how this should be implemented is still often debated. There have been numerous publications on this topic in the past but this still remains a hot discussion point amongst Data Managers.

This presentation will review the aims of data checking, the pros and cons of doing it; when and how it should be done (i.e. real time, end of study or periodically); what it does and what it doesn't do; and the opinions of regulators and trialists both historically and currently. If data entry checking is performed what is an acceptable error rate? Does rigorous data checking have any impact on your analysis? Is there any evidence for this?

We will also review how the requirements have changed with the increasing use of electronic data collection and how to manage the process if you are using a hybrid of paper and electronic systems. We will examine how the requirements may differ for small single centre studies, large multi-centre studies and international studies where there may be training or language barriers. Consideration will be given to different types of data entry operators from site staff, professional data entry clerks and patients entering data online. Real-life examples will be used to illustrate the points being discussed.

